# Statistical classification of treatment responses in mouse clinical trials for stratified medicine in oncology drug discovery

**DOI:** 10.1038/s41598-023-51055-7

**Published:** 2024-01-09

**Authors:** Hélène Savel, Florence Meyer-Losic, Cécile Proust-Lima, Laura Richert

**Affiliations:** 1 U1219, Inserm Bordeaux Population Health Research Centre, Department of Public Health, Université de Bordeaux, 33000 Bordeaux, France; 2 Ipsen Innovation, 5 Avenue du Canada, 91940 Les Ulis, France; 3 Institut Bergonié, CHU de Bordeaux, INSERM, Université de Bordeaux, CIC-EC 1401, 33000 Bordeaux, France; 4 Inria, SISTM, 33400 Talence, France; 5 Université de Bordeaux, 146 Rue Léo Saignat, 33076 Bordeaux Cedex, France

**Keywords:** Cancer, Computational biology and bioinformatics, Drug discovery

## Abstract

Translational oncology research strives to explore a new aspect: identifying subgroups that exhibit treatment response even during pre-clinical phases. In this study, we focus on PDX models and their implementation in mouse clinical trials (MCT). Our primary objective was to identify subgroups with different treatment responses using Latent Class Mixed Model (LCMM).We used a public dataset and focused on one treatment, encorafenib, and two indications, melanoma and colorectal cancer, for which efficacy depends on a specific mutation BRAF V600E. One LCMM per indication was implemented to classify treatment responses at the PDX level, analyzing the growth kinetics of treated tumors and matched controls within the PDX models. A simulation study was carried out to explore the performance of LCMM in this context. For both applications, LCMM identified classes for which the higher the proportion of mutated BRAF V600E PDX models the greater the treatment effect, which is aligned with encorafenib use recommendations. The simulation study showed that LCMM could identify classes with large differences in treatment effects. LCMM is a suitable tool for MCT to explore treatment response subgroups of PDX. Once these subgroups are defined, characterization of their phenotypes/genotypes could be performed to explore treatment response predictors.

## Introduction

Oncology research is increasingly shifting towards stratified medicine, aiming to provide each patient with the most appropriate treatment based on their pre-treatment (baseline) characteristics^1,2^. In certain cancer types and treatments, patient responses to treatment can vary significantly, ranging from a complete response to disease progression, often influenced by specific molecular characteristics of the cancer. For instance, in melanoma and colorectal cancer and with encorafenib treatment, the presence of baseline BRAF V600E mutations has been identified as a predictor of treatment response^3–8^. However, when developing a new drug, the predictive factors for treatment response may not yet be known. In such cases, early exploration of patient subgroups with distinct treatment responses and assessment of their baseline predictors can optimize the drug development process, allowing for a targeted focus on relevant subgroups from the outset, even during preclinical phases for drug discovery research^9^.

**Figure 1:**
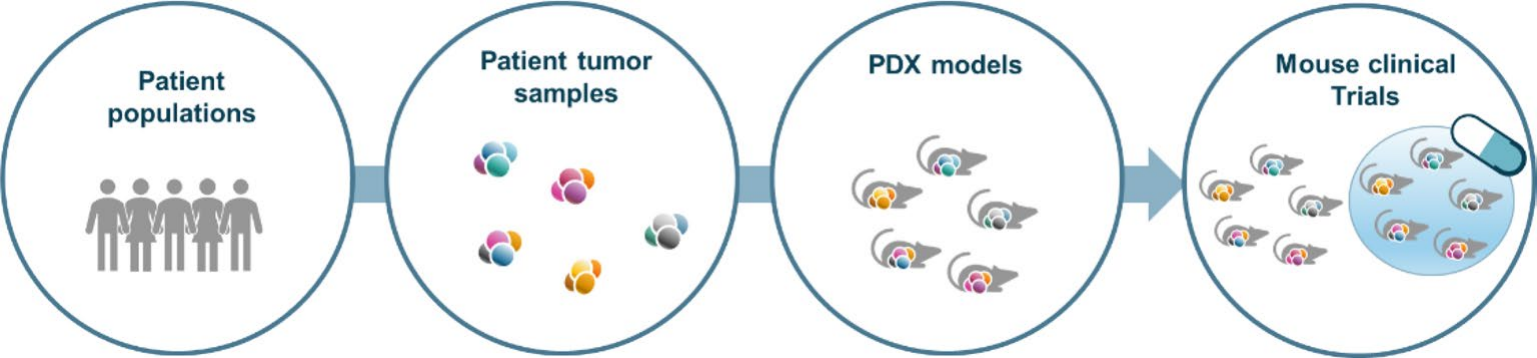
Illustration of the concept of a Mouse Clinical Trial.

In preclinical oncology research, Mouse Clinical trials (MCT) or PDX Clinical trials (PCT, and PDX for Patient Derived Xenograft)^10,11^ are experimental approaches that enable the incorporation of patient heterogeneity in the drug development path. Patient-Derived Xenografts (PDX) are created by implanting tumors from patients in immunocompromised mice and are known to retain key characteristics of the original patient tumor. In MCT, each PDX model represents a tumor from a different patient, and within each PDX, mice are randomly assigned to a vehicle control group or various treatment arms. Therefore, mice within the same PDX model have an identical tumor, while tumors differ across PDX models. A MCT involves multiple PDX models, allowing observation of treatment responses from different models in each treatment arm as described in Fig. 1. Similar to clinical trials, including multiple PDX models (each with a tumor from a different patient) in the MCT captures the heterogeneity present in derived patient tumors, with greater diversity captured as the number of PDX models increases.

The exploration of predictive characteristics of treatment response in PDX models involves two steps. The first step is to classify treatment responses and identify subgroups of PDX models that respond differently to treatment. The second is to assess the baseline molecular signatures that could distinguish responders and non-responder subgroups. Our work focuses on the first step, i.e. on methods for classifying treatment responses.

In the literature, some authors such as Gao et al.^12^ have developed a mouse-specific ordinal response definition called mRECIST, which categorizes treatment responses as complete response, partial response, stable disease or progressive disease based solely on the kinetics of each treated mouse. However, this method disregards information from untreated control mice, which reflects the natural tumor progression and thus prognosis. Consequently, the mRECIST approach does not specifically assess causal treatment effects but describes outcomes that are likely confounded by natural prognosis. Another criterion used in the literature, Tumor Growth Inhibition (TGI = 1 − RTV_tr_/RTV_c_, with Relative Tumor Volume RTV = TV_t_/TV_0_, t: time, 0: randomization date, tr: treated and c: control), takes into account the control group by forming a ratio of relative tumor volume between treated and control mice^10^. However, there is no consensus on the threshold for this criterion, and it often leads to suboptimal statistical power as it does not fully consider the complete data on tumor growth kinetics. Laajala et al.^13^ developed a model for analyzing preclinical oncology data using mixed-effect regression models, classifying models into “growing” and “poorly growing” categories.

To address the limitation of existing methods for classifying tumor growth kinetics in PDX, we propose the use of Latent Class Mixed Models (LCMM). In epidemiology, this model is employed when acknowledging heterogeneity in the trajectories of a biomarker and aiming to identify and characterize classes of trajectories large cohorts. These classes are referred to as latent classes because the model assumes that the observed heterogeneity is a result of unobservable variables. For instance, it has been applied in the study of cognitive decline^14^. In a previous work, we demonstrated that linear and non-linear mixed-effects models could robustly estimate treatment effect in a MCT design^15^. Therefore, our objective was to explore the performances of the LCMM method to classify treatment trajectories in PDX models.

## Materials and methods

The Latent Class Mixed Model (LCMM) was used to distinguish treatment response subgroups, defined by tumor growth kinetics of control and treated mice. To consider the classification of both the kinetics of the treated mouse and the associated control mouse, the model was defined at the PDX level rather than the mouse level.

### MCT datasets

To test the ability of the LCMM model to explore heterogeneity in a MCT we focused on encorafenib treatment in two indications: melanoma and colorectal cancer. This choice was made because differences in treatment responses are expected in this treatment-indication combination, and a mutation predictive of treatment (BRAF V600E) is already known, thus making these datasets suitable for the proof-of concept of our classification method. This mutation information was not taken into account in the modelling approach, since we propose LCMM as a method suitable for the exploration of responder subgroups in the absence of such prior knowledge. However, we used this mutation information to assess the biological plausibility of the resulting classification. The dataset used was the open access dataset associated with the publication by Gao et al.^12^. One untreated mouse and one mouse per studied treatment were included in each PDX model, and 33 PDX were included in the MCT. Treatment was administered for 21 days at the reverse translated from human dose. For this analysis, we focused on the first 28 days of follow-up. Details of the experiment are described in the publication^12^. The data reported by Gao et al. used for the analysis were the tumor volume measurements and the time of measurement during the first 28 days of follow-up for each mouse, and the BRAF V600E mutation status for each PDX model.

### Latent class mixed model

In contrast to standard mixed effect regression models which assume the trajectories of the marker under study come from a homogeneous population, latent class mixed models assume the population is heterogeneous and characterized by several distinct mean profiles of trajectory. This is achieved by defining an unobserved class structure with each individual or experimental unit belonging to one and only one class. In the case of MCTs, our aim was not to identify distinct profiles of individual trajectories at the mouse level but to identify distinct causal treatment effects at the PDX level. By defining the PDX as the experimental unit, we were able to take into account both the control and treated mouse within each PDX and define classes with different treatment effects in terms of effect sizes between treated and untreated groups, rather than identifying only classes with different baseline prognosis.

The LCMM model was defined by two statistical sub-models as follows:

Let c_i_ be a discrete latent variable for the latent group structure: c_i_ = g if the experimental unit (in our case PDX) i (i = 1,…,N) belongs to class g (g = 1,…,G).

- Probability of latent class membership described using a multinomial logistic model defined as follows:
  $${\pi }_{ig}=P\left({c}_{i}=g|{X}_{ci}\right)=\frac{{e}^{{\xi }_{0g}+{X}_{ci}{\prime}{\xi }_{1g}}}{{\sum }_{l=1}^{G}{e}^{{\xi }_{0l}+{X}_{ci}{\prime}{\xi }_{1l}}}$$
  where ${\xi }_{0g}$ is the intercept for the class g and ${\xi }_{1g}$ is the q_1_-vector of class specific parameters associated with the q_1_-vector of time-independent covariates X_ci_.

With ${\xi }_{0G}=0 \text{ and } {\xi }_{1G}=0$ i.e. class G is the reference class. When no covariate predicts the latent class membership, this model reduces to a class-specific probability, which is the case in our application.

- Class-specific trajectory of the marker defined by a linear mixed model

In the context of MCTs, it is assumed that for the same PDX model, there is no individual mouse effect since mice are clones. We assumed the difference in tumor growth between the treated and the control mice was only due to the treatment. Under this assumption, the class-specific linear mixed model was defined for tumor volume (in log) Y_ijk_ measured for each PDX i = 1,…, N, mouse k = 1, 2 and occasion j = 1, …, n_i_ corresponding to time t_ijk_ (number of tumor volume measurements for each mouse) as follows:

$${Y}_{ijk}{|({c}_{i}=g) \sim \beta }_{0g}+{\beta }_{1g}f\left({t}_{ijk}\right)+{\beta }_{2g}{Trt}_{ik}+{\beta }_{3g}f\left({t}_{ijk}\right){Trt}_{ik}+{\gamma }_{0ig}+{\gamma }_{1ig}f\left({t}_{ijk}\right)+{\varepsilon }_{ijk}$$

$${\gamma }_{i}|({c}_{i}=g)=\left(\begin{array}{c}{\gamma }_{0ig}\\ {\gamma }_{1ig}\end{array}\right)\sim N\left(\left[\begin{array}{c}0\\ 0\end{array}\right],\left[\begin{array}{cc}{\sigma }_{{\text{g}}}^{2}& {\sigma }_{{\gamma }_{0g}{\gamma }_{1g}}\\ {\sigma }_{{\gamma }_{0g}{\gamma }_{1g}}& {\sigma }_{{\text{g}}}^{2}\end{array}\right]\right)$$

$${\varepsilon }_{ijk} \sim N\left(0,{\sigma }_{\varepsilon }^{2}\right)$$

$$\left({\gamma }_{1g}\dots {\gamma }_{Ng}\right) \bot \left({\varepsilon }_{1}\dots {\varepsilon }_{N}\right)$$

where f(t)=${\alpha }_{1}t+{\alpha }_{2}({1}_{t>8}) (t-8)$ is the function of time capturing the overall shape of trajectory (here two linear slopes with a change point at days 8), trt is the treatment group (treated vs control coded 1/0) and ${\gamma }_{i}$ are the individual random effects that capture the correlation in the PDX repeated measures of the outcome.

The estimation of the parameters was carried out in the maximum likelihood framework using R package lcmm^14^ in the R software version 4.1.0 (R Project for Statistical Computing, RRID:SCR_001905).

In LCMM models, the number of classes is not directly estimated. Models with 2, 3 and 4 classes were estimated, and the final number of latent classes was chosen based on the Integrated Classification Log-likelihood criterion (ICL-the lower the better) which combines the Bayesian Information Criterion (measure of the adequacy of the model to the data) and the discrimination of the classes (with a measure derived from the posterior probabilities of latent class membership). Given the small sample size, the selection of the final number of latent classes was also guided by the parsimony of the model. Once the optimal model was identified, the second step was to describe, within each class, the tumor growth trajectories of the treated and control mice. A classification was also derived from the model by assigning a PDX to the class in which it had the highest posterior probability (i.e., the class membership probability in the estimated model given the PDX observations) to belong.

Of note, to ensure a convergence toward the global likelihood maximum, each model was estimated 300 times from different random initial values.

### Simulations

A simulation study was conducted to explore the performance of LCMM for treatment effect classification (defined by both treated and untreated tumor growth kinetics) in the context of MCTs, i.e. when considering a small number of experimental units (a few dozen) as opposed to its classic use with hundreds or thousands individuals in epidemiology.

Nine scenarios were simulated with three different patterns of treatment effect differences between the two classes (− 1, − 2.5 and − 5 log(mm^3^)/days), and three different intensities of random slope variability (at the 10th, 50th and 90th percentiles of the random slopes estimated using the mixed linear regression models by indication and treatment in the public data from Gao et al.^12^). The kinetic profiles of these 9 scenarios are detailed in supplementary Fig. 1. In these simulations, we assumed two mice (one treated and one untreated mouse) per PDX model with a 28-day follow up and several sample sizes of PDX models (N = 20 and 40). For each scenario, 500 simulated datasets were generated. Tumor growth kinetics were simulated using a two classes mixed model with a class-specific linear trajectory, a class-specific treatment effect and individual random intercept and slope^15^. These datasets were then analyzed with a 2-class LCMM model as described in a previous section. To assess the accuracy of the resulting classification, a percentage of mismatch between the generated classes and the classes obtained by the estimated LCMM was defined as follows:

$$\%mismatch=\frac{{n}_{error}}{N}\times 100$$

With N: the number of PDX models in the MCT; And n_error_: the number of PDX differently classified in the generated and estimated LCMM models.

## Results

### Classification of encorafenib treatment effect in melanoma and colorectal cancer MCT

LCMM models with 2 to 4 latent classes were estimated for each indication, specifically melanoma and CRC, and all models converged successfully.

#### Melanoma use case

Based on the ICL criterion, the model with the lowest ICL was the 4-class LCMM model (2-class LCMM: ICL = 4994.2, 3-class LCMM: ICL = 4858.2, 4-class LCMM: ICL = 4773.2). However, the 3-class LCMM had an ICL value close to the one of the 4-class model, and one class of the 4-class model contained only one PDX. Additionally, the assignment in each class was associated with posterior probabilities of almost 1 in the 3-class model, indicating its extremely high level of separation. We thus retained the 3-class LCMM.

**Figure 2:**
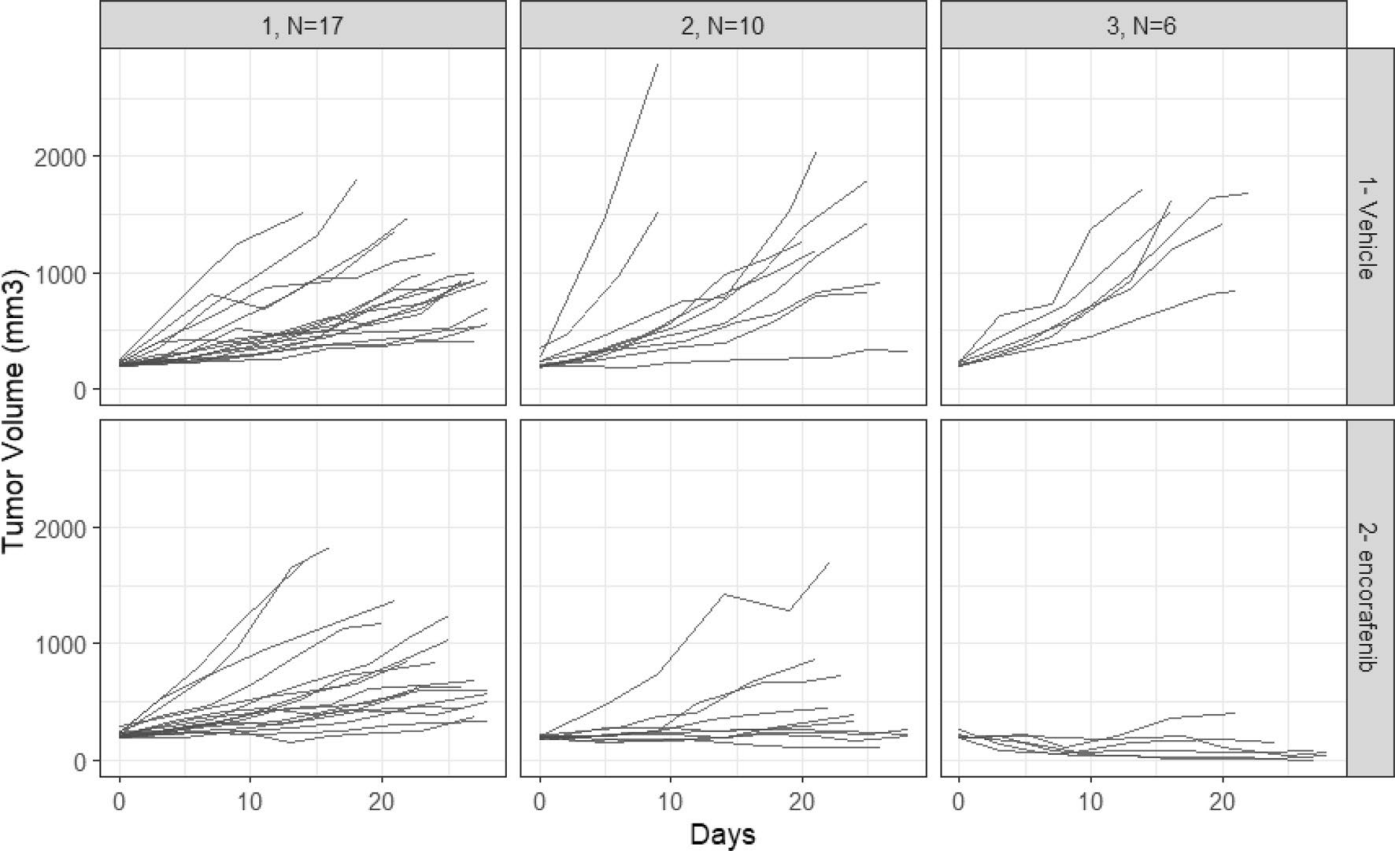
Classification of tumor growth kinetics of melanoma PDX models in each treatment group as determined by the LCMM.

**Figure 3:**
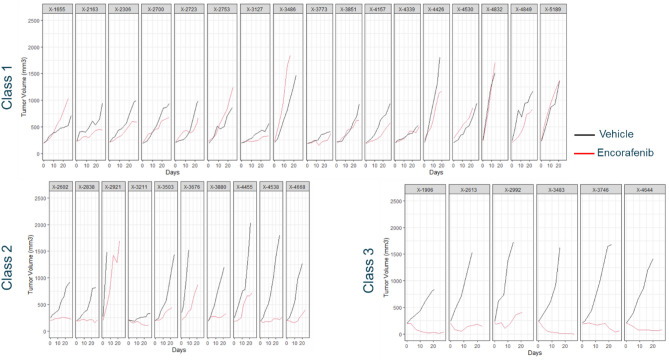
Melanoma PDX tumor growth kinetics in each latent class identified by the LCMM.

The tumor growth kinetics of PDX in each assigned class are reported by treatment in Fig. 2, and by PDX in Fig. 3.

There was no treatment effect observed in class1, a weak treatment effect in class 2 and a larger treatment effect in class 3.

The biological plausibility of the classification was tested based on the available baseline mutation information. The overall proportion and associated 95% confidence interval of mutated BRAF V600E PDX models were 36.4% [20.4; 54.9]. The proportion and associated 95% confidence interval of BRAF V600E mutated PDX models were 17.6% [3.8; 43.4], 40% [12.1; 73.8] and 83.3% [35.9; 99.6] in classes 1, 2, 3, respectively (Fisher exact test, p value = 0.015). The LCMM derived classification was thus in line with the biological existing knowledge of treatment effect predictors for encorafenib, where the treatment is effective in patients with a BRAF V600E mutation^6,7^.

#### Colorectal cancer use case

For the colorectal cancer use case, based on the ICL criterion, the model with the lowest ICL was the 4-class LCMM model (2-class LCMM: ICL = 5965.1, 3-class LCMM: ICL = 5827.5, 4-class LCMM: ICL = 5751.4). However, the 3-class model showed already an almost perfect separation of classes with mean posterior class-membership probabilities > 0.97. Considering these criteria and giving preference to the more parsimonious adequate class number, the 3-class LCMM model was retained.

**Figure 4:**
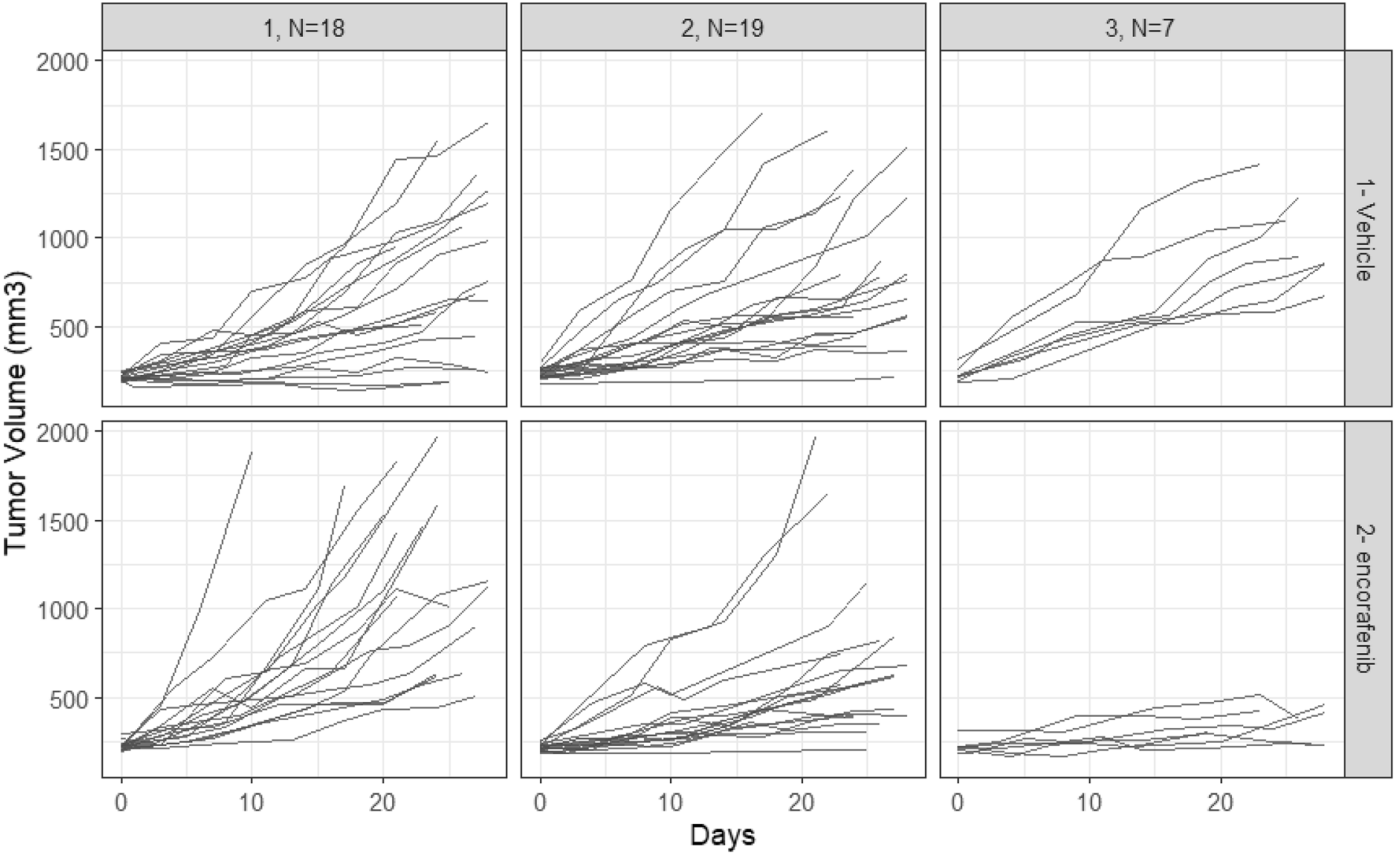
Classification of tumor growth kinetics of CRC PDX models in each treatment group as determined by the LCMM.

**Figure 5:**
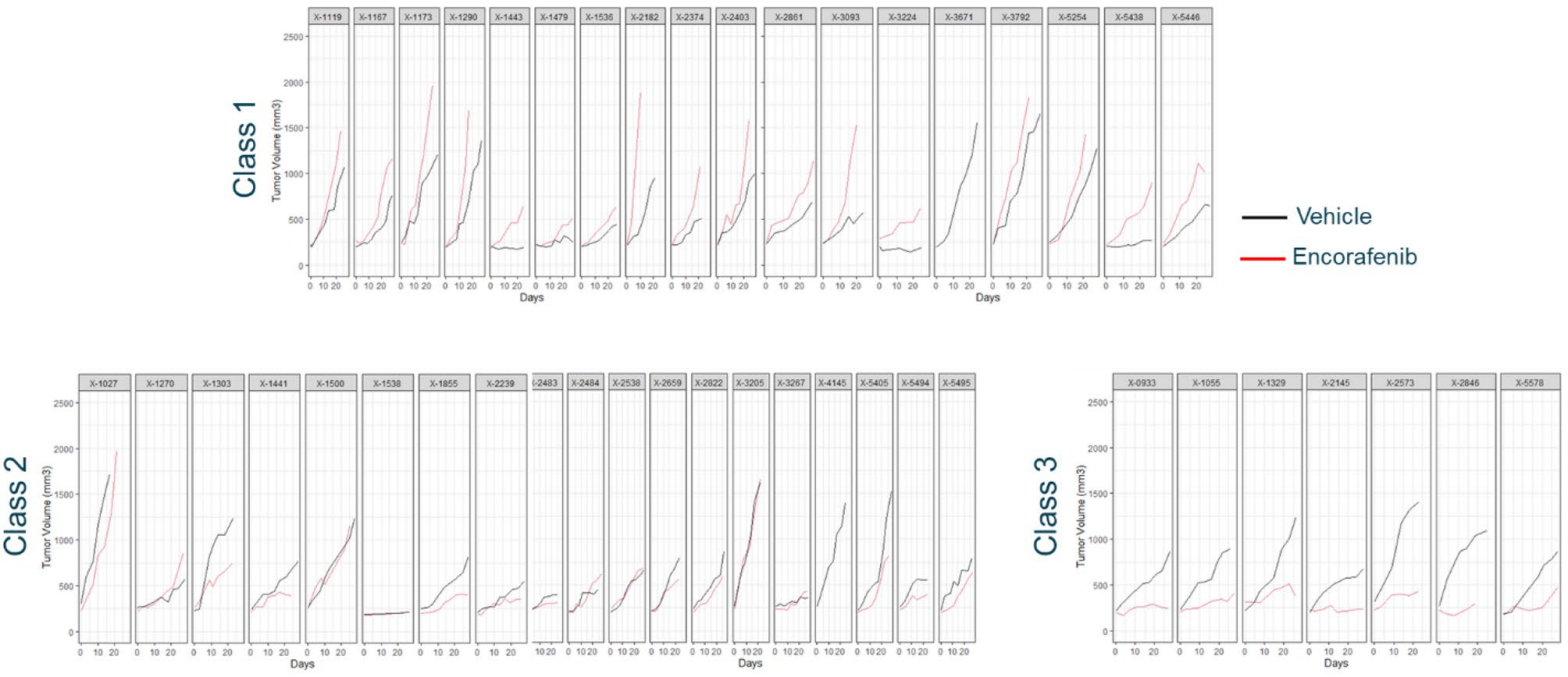
CRC PDX tumor growth kinetics in each latent class identified by the LCMM.

The reported tumor growth kinetics per assigned classes (Figs. 4, 5) suggest that there was a deleterious treatment effect in class 1, no treatment effect in class 2 and a treatment effect in class 3. In this MCT, the overall proportion and associated 95% confidence interval of BRAF V600E mutated PDX models were 13.6% [5.2; 27.3]. The proportions and associated 95% confidence interval of BRAF V600E mutated PDX models per class were 5.2% [0.1; 26.0], 16.7% [3.6; 41.4] and 28.6% [3.7; 70.9] in classes 2, 1, 3 respectively. The frequency of BRAF V600E mutated PDX models tended to be higher in the responder class (class 3) compared to the non-responder classes (class 1 and class 2) although this did not reach statistical significance (Fisher exact test, p value = 0.19). The LCMM derived classification was thus in line with the biological existing knowledge of treatment effect predictors for encorafenib^3–5,8^.

### Simulations

**Figure 6:**
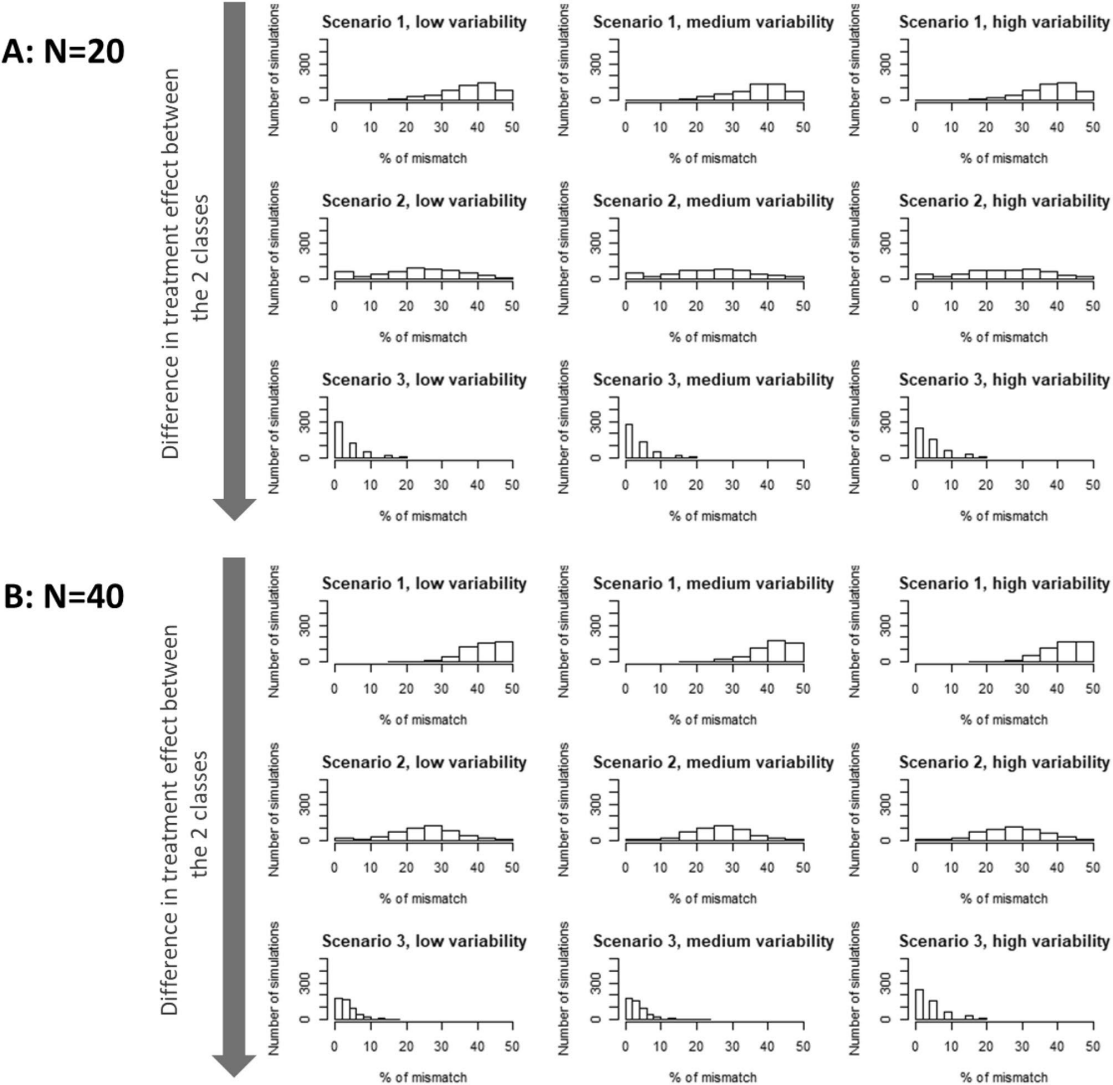
Description of the proportion of mismatch of the simulation study. Scenarios are defined according to different values of difference in treatment effect between the 2 classes (− 1 log(mm^3^)/day for scenario 1, − 2.5 log(mm^3^)/day for scenario 2 and − 5 log(mm^3^)/day for scenario 3). Low, medium and high variability correspond to the 10th, 50th and 90th percentile of the random slopes of linear mixed models (4.5, 11.5 and 32.2 respectively) by indication and treatment from a real dataset of 6 indications and at least 10 treatments per indication^12^.

Results of the simulation study are reported in Fig. 6. They indicate that, for two realistic sample sizes of MCT (N = 20 or 40 PDX), with different levels of inter-PDX variability of tumor growth (random slope), the greater the difference in treatment effect between classes, the better the classification of treatment response derived from LCMM. In view of these results, applying this method to a real dataset can allow identifying classes in cases of large treatment effect differences between classes. This result is in line with one of the translational research objectives which is to identify responders to treatment models versus non-responders that have by definition a significant difference of treatment effect.

## Discussion

In MCTs, identifying subgroups of PDX models with a treatment response is a major challenge. Therefore, an informed choice of the most appropriate statistical method is of importance to reduce the risk of bias in estimating treatment effect and to have an objective definition of treatment responders. The LCMM allows for the classification of tumor growth kinetics, considering both the control and treated groups (reducing the risk of bias in estimating treatment effect), without requiring prior knowledge of the kinetic profile. The application of this model on two specific use cases successfully distinguished treatment responses. Furthermore, the resulting classification was plausible in terms of prior biological knowledge indicating that treatment response varies depending on BRAF V600E mutation^3–8^.

The simulation study showed that the use of LCMM can only distinguish classes with a large difference in treatment effect in the context of small sample sizes in MCTs. Indeed, the greater the difference in treatment effect between classes, the lower the percentage of mismatch. In the preclinical MCT application, the LCMM is used in the context of drug discovery research, with the aim of identifying subgroups of treatment responders (with a significant treatment effect) vs. non-responders (with no treatment effect). To be of interest for decision making in the development plan, these two subgroups should have a large difference in treatment effect, which is in line with the results of the simulation study and supports the value of using LCMM to classify tumor growth kinetics of PDX models.

After identifying the treatment-response classes using a LCMM model and a biological interpretation of the results, further bioinformatics and data science analyses could be conducted to discover baseline tumor signatures distinguishing the classes, such as their phenotypes or genotypes. If such baseline signatures, driving the treatment response, were discovered, these could be considered in the clinical development plan, for instance by selecting subgroups of patients with the highest likelihood of responding to the treatment in early phase trials or by designing trials specifically to assess subgroup treatment effects in human participants as basket trial or umbrella trial designs^16–18^. If no classes or no associated baseline signatures were discovered in the MCT, exploration of potential subgroup treatment effects could nevertheless be pursued in the clinical phases.

In conclusion, in the context of MCTs, LCMM is a suitable statistical method for identifying subgroups of PDX models with large differences in treatment effects. This contributes to the aim of better selecting patients with the best chance of responding to treatment in early phase clinical trials.

### Supplementary Information

Supplementary Information.

## Data Availability

Data used for this analysis are available in the supplementary data associated with Gao et al.^12^ publication. Qualified researchers may request access to the computer codes used to generate the simulated data by contacting the first author of this manuscript.
