# Supplementary material for: Statistical classification of treatment responses in mouse clinical trials for stratified medicine in oncology drug discovery
Source: Sci Rep. 2024 Jan 9;14:934. doi: 10.1038/s41598-023-51055-7 (PMC10776864; doi:10.1038/s41598-023-51055-7)

**Supplementary figure 1: Description of the kinetic profiles of the nine simulation scenarios of the simulation study.**

Scenarios are defined according to different values of difference in treatment effect between the 2 classes (-1 log(mm^3^ )/day for scenario 1, -2.5 log(mm^3^ )/day for scenario 2 and -5 log(mm^3^ )/day for scenario 3). Low, medium and high variability correspond to the 10^th^, 50^th^ and 90^th^ percentile of the random slopes of linear mixed models (4.5, 11,5 and 32.2 respectively) by indication and treatment from a real dataset of 6 indications and at least 10 treatments per indication (Gao et al., 2015) **.**


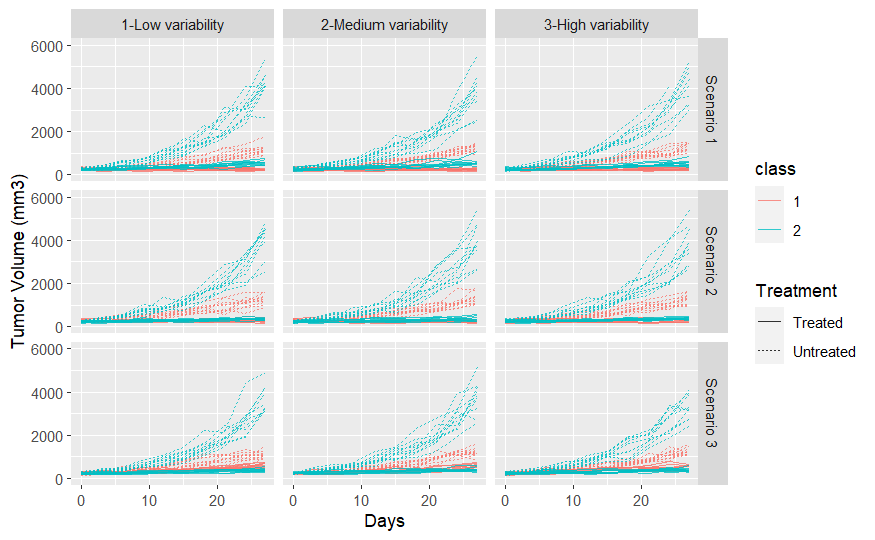

Supplement: Supplementary file 1 — Supplementary Information. [file 41598_2023_51055_MOESM1_ESM.docx]
